# Near-IR Absorbers Based on Pt(II)-Dithiolene Donor–Acceptor Charge-Transfer (CT) Systems: A Structural Analysis to Highlight DA Interactions

**DOI:** 10.3390/molecules28062566

**Published:** 2023-03-11

**Authors:** Davide Espa, Luca Pilia, Flavia Artizzu, Angela Serpe, Paola Deplano, Luciano Marchiò

**Affiliations:** 1 Dipartimento di Scienze Chimiche e Geologiche, Università di Cagliari, Unità di Ricerca dell’INSTM, S.S. 554-Bivio per Sestu, 09042 Cagliari, Italy; 2 Dipartimento di Ingegneria Meccanica Chimica e dei Materiali, Università di Cagliari, Via Marengo 2, 09123 Cagliari, Italy; 3 Dipartimento per lo Sviluppo Sostenibile e la Transizione Ecologica (DiSSTE), Università del Piemonte Orientale “A. Avogadro”, Piazza S. Eusebio 5, 13100 Vercelli, Italy; 4 Dipartimento di Ingegneria Civile, Ambientale e Architettura, INSTM Research Unit, Università di Cagliari, Via Marengo 2, 09123 Cagliari, Italy; 5 Istituto di Geologia Ambientale e Geoingegneria, Consiglio Nazionale delle Ricerche (IGAG-CNR), Via Marengo 2, 09123 Cagliari, Italy; 6 Dipartimento di Scienze Chimiche della Vita e della Sostenibilità Ambientale, Università di Parma, Parco Area delle Scienze 17/a, 43124 Parma, Italy

**Keywords:** near-IR absorbers, charge transfer, donor–acceptor, stacking, Hirshfeld analysis, Pt-dithiolenes

## Abstract

The packing interactions of a series of electron donor (D) and electron acceptor (A) charge transfer (CT) near-IR absorbers based on platinum-dithiolene complexes are reinvestigated here as a case study also by using the Hirshfeld surface analysis. This analysis on systems, which exhibit the 1:1, 2:1 and 2:2 columnar stacking patterns between D and A, allows us to point out that several interactions of atoms and fragments are involved in the stacking interactions but also that only a limited fraction of these interactions, limited to the 1:1 D/A columnar stacking case, can be relatable to the absorption features of this class of compounds.

## 1. Introduction

**Scheme 1:**
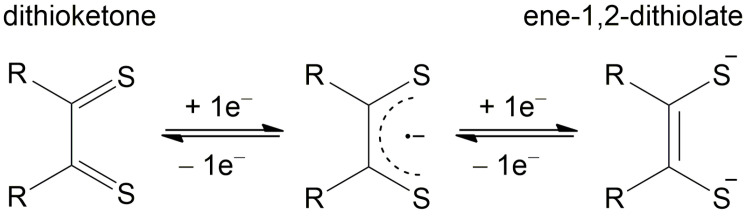
Different formal oxidation states of dithiolene ligands.

The dithiolene complexes of transition metals are a class of covalent compounds with a number of unusual properties related to the peculiar nature of the bonding [1]. In these complexes, the term dithiolene is employed irrespective of the real form of the ligand, which can bind a variety of metals as neutral dithioketone, mixed-valence thioketone-radical thiolate monoanion and ene-1,2-dithiolate dianion (A, B, C in Scheme 1, respectively).

**Scheme 2:**
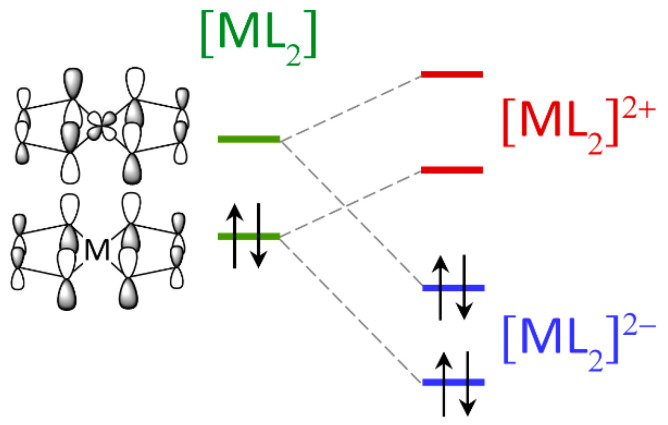
Frontiers Molecular Orbitals for [ML_2_] compounds whose energies are pushed up [ML_2_]^2+^ or down [ML_2_]^2−^ by strong π-donor or strong π–acceptor substituent at the dithiolene core, respectively. Accordingly, [ML_2_]^2+^ or [ML_2_]^2−^ can be isolated.

In d^8^-metal dithiolene compounds, the metal atom imposes the planarity of the central core, whereas the electronic properties depend on the distribution of the π-electrons in the (C_2_S_2_)_2_M system yielding a sort of ‘aromaticity’ [2]. The nature of the substituents at the C_2_S_2_ moieties affects the energy of the frontier orbitals where a significant mixing between the metal- and ligand-centered orbitals occurs, as shown in Scheme 2.

The HOMO–LUMO transition in neutral complexes gives rise to an intense electronic transition at unusually low energies, and the related broad peak does not show vibrational fine structure, as occurs with organic dyes, such as the cyanines. Since the early 1990s, Mueller-Westerhoff highlighted the high potential of these complexes as NIR-dyes [3] tunable with the nature of substituents at the C_2_S_2_ moiety. Optical properties, combined with their thermal stability, have allowed their employment for a variety of applications including in Q-switching infrared lasers. More recently, dithiolene-dyes have been exploited in Photo Thermal Therapy (PTT) [4,5] and also proposed for bioimaging [6] by using the monoreduced 1,3-diisopropylimidazoline-2,4,5-trithione as a ligand, which is capable of providing dyes absorbing near 1000 nm, and with absorptivities falling in the range of the highest values so far achieved [7].

Moreover, heteroleptic dithiolene derivatives such as bipyridine/dithiolate Pt(II) square-planar complexes have been shown to be suitable precursors of NIR-dyes. The optical properties and related applications of these complexes have been widely investigated [8,9,10]. In these complexes, which display an asymmetric charge distribution, the HOMO has mixed contributions from both Pt and dithiolate orbitals, while the LUMO is diimine ligand based. Accordingly, the HOMO–LUMO transition has a charge-transfer character (mixed metal−ligand-to-ligandcharge transfer: MMLL′CT) and gives rise to absorption peaks falling in the visible region at wavelengths that are tunable with the electro-donating–accepting nature of the ligands.

**Scheme 3:**
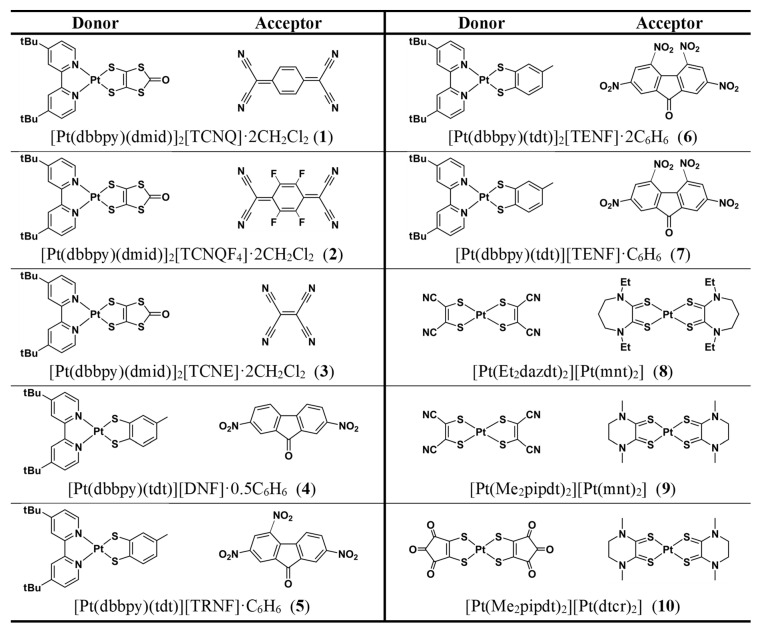
Molecular structures of the compounds discussed in the work. dbbpy = 4,4′-di-tertbutyl-2,2′-bipyridine; tdt = 3,4-toluenedithiolate; dmecb = 4,4′-dimethoxyester-2,2′-bipyridine; dmid = 2-oxo-1,3-dithiole-4,5-dithiolate; Me_2_pipdt = *N*,*N*′-dimethyl-piperazine-2,3-dithione; Et_2_dazdt = *N*,*N*′-diethyl-perhydrodiazepine-2,3-dithione; mnt = maleonitrile-2,3-dithiolate; dtcr = 4,5-disulfanylcyclopent-4-ene-1,2,3-trionate, known as dithiocroconate.

In addition, bipyridine/dithiolate Pt(II) square planar complexes have been employed as donors towards acceptors based on tetranitrile and nitrofluorenone system as acceptors [11] for the production of the so-called thin-film organic photovoltaic (OPV) devices [12,13,14,15,16]. OPVs comprise a molecular donor (D) in the form of a thin film (e.g., copper phthalocyanine, CuPc) together with a molecular acceptor (A) (e.g., fullerene), which interact in alternate or mixed layers. With the view to improve the properties of OPV devices by employing adducts with a better overlap of the absorption spectrum with the solar radiation Dunbar, Omary et al. [17], have deeply investigated the properties of the adducts **1**–**3**, obtained by combining [Pt(dbbpy)(dmid)] with a planar tetranitrile system and adducts **4**–**7** obtained by combining [Pt(dbbpy)(tdt)] with nitrofluorenone derivatives (see compounds in Scheme 3). An increase in absorbance across the visible and into the NIR region with respect that of the isolated donor and acceptors was observed in the adducts, but the desirable additional band into the NIR region at lower energy (with respect that of the isolated donor and acceptors) was observed only for adducts **5** and **7**.

Compounds **8**–**10** in Scheme 3 represent a third class of D-A CT adducts, which show an additional NIR peak as compared to the donor and acceptor components [18,19,20,21,22,23,24,25]. Both the donor and the acceptor are based on d^8^-metal-dithiolenes [metal = Pd(II) or Pt(II)] with different charges (+2 and −2; see Scheme 2), associated with the nature of the substituents at the C_2_S_2_ moieties. In fact, taking into account the neutral form, the strong π-donor substituents close to the C_2_S_2_ (Me_2_pipdt and Et_2_dazdt) raise the energy of the frontier orbitals, thus yielding [ML_2_]^2+^ cationic complexes. In contrast, strong π-acceptor substituents (mnt and dtcr) lower the energies of the frontier orbitals, thus favoring the [ML_2_]^2−^ anionic state (see Scheme 2). Additionally, CT salts of the type {(C^2+^)[ML_2_]^2–^}, (M = Ni, Pd, Pt; L^2–^ = dithiolato; C^2+^ = 4,4′ and 2,2′ bipyridinium derivatives) previously investigated by Kisch et al. [26,27] exhibit a strong NIR additional band with respect to the components in the solid state.

It seemed interesting to us to revisit the crystal packing features and the interactions occurring between the components in **1**–**10** reported in Scheme 3, as a case study and also by means of the Hirshfeld surface (HS) analysis. Our aim is to evaluate the non-covalent interactions controlling the self-assembly [28,29], to point out the factors which favor or disfavor the presence of an NIR additional band for the adduct with respect to the isolated donor and acceptor.

## 2. Results and Discussion

### 2.1. Solid-State NIR Electronic Absorption Spectra and Electrochemical Properties

As shown in Scheme 3, the donor [Pt(dbbpy)(dmid)] (dbbpy = 4,4′-di-tertbutyl-2,2′-bipyridine; dmid = 2-oxo-1,3-dithiole-4,5-dithiolate) is combined with the acceptors based on a planar tetranitrile system (TCNQ, TCNQF_4_ and TCNE), to form **1**, **2** and **3** adducts as dark crystals. These adducts exhibited a 2:1 D/A ratio, even when a 1:1 D/A ratio was experimentally employed. In the diffuse reflectance spectra, the isolated donors exhibit the predictable MMLL′CT peaks in the 550–850 nm region. Moreover, in the **1**, **2** and **3** adducts, no additional bands associated with the donor–acceptor (DA) CT at lower energy are observed, but only an increase in absorbance across the visible and into the NIR region with respect that of the isolated donor and acceptors (π→π*). The lack of DACT can be explained considering that the compounds **1**–**3** show a 2:1 donor–acceptor stacking pattern, whereas it was established that a 1:1 DA arrangement is required to observe DACT absorption bands [26,27]. Despite the absence of a DACT, but considering the continuous absorption ranging from the UV to the NIR, these adducts were also tested as dyes in a TiO_2_ solar cell, and low conversion efficiencies were obtained.

**Figure 1:**
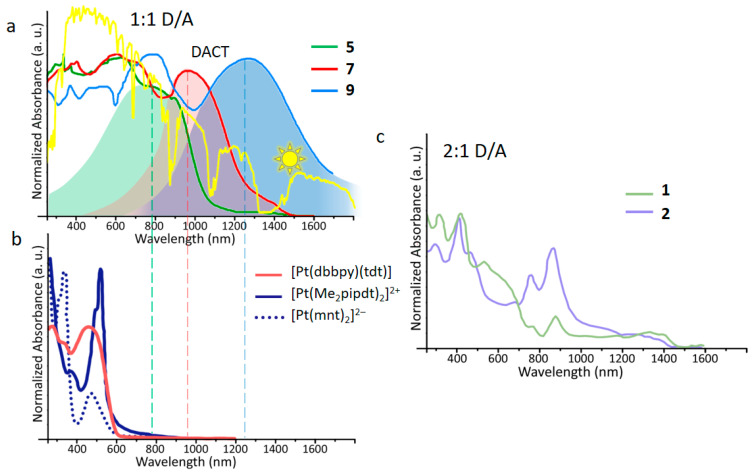
Normalized diffuse reflectance spectra of crystalline samples of: (**a**) **5** (green), **7** (red) and **9** (blue), evidencing the DACT band (shadowed area) in the NIR and the overlap with the AM1 solar spectrum (yellow); (**b**) [Pt(dbbpy)(tdt)], donor unit of **5** and **7** (red), [Pt(mnt)_2_]^2−^ (blue dotted line) and [Pt(Me_2_pipdt)_2_]^2+^, donor and acceptor (blue solid line) units of **9**, respectively; (**c**) **1** and **2**, displaying no absorption band attributable to DACT in the NIR. Dashed lines are a guide to the eye.

The adducts **4**, **5**, **6** and **7** comprise [Pt(dbbpy)(tdt)] as donor (tdt = 3,4-toluenedithiolate), whereas the acceptors are increasingly strong nitrofluorenone moieties: 2,7-dinitro-9-fluorenone (DNF), 2,4,7-trinitro-9-fluorenone (TRNF), or 2,4,5,7-tetranitro-9-fluorenone (TENF). In **4**, **5**, **6** and **7**, a 2:1 ratio of D/A molecules was employed to obtain the adducts (CH_2_Cl_2_ solutions of the donor with solutions of the organic acceptor in a CH_2_Cl_2_/C_6_H_6_ solution). Despite the experimental molar ratio adopted, adducts with a 1:1 D/A ratio were isolated in **4** and **5**, while a 2:1 D/A ratio was invariably obtained in **6**. Against this, **7** was obtained only when a 1:1 [Pt(dbbpy)tdt]:TENF ratio was used. No additional band with respect to the components was observed in **4** both in solution and in the solid state. Instead, the diffuse reflectance spectra show the presence of an additional band with respect to those of the components for **5** (825nm) and **7** (950 nm, see Figure 1). These bands, which become evident also in solution by the addition of TRNF or TENF to [Pt(dbbpy)(tdt)] falling in an NIR region free of peaks related to the reagents, are assigned to the DACT transition.

Single crystal results have shown that the 1:1 D/A combination ratio gives rise to crystals with a 1:1 D/A stacking only for **5** and **7** (see related section), while a 2:2 -DDAA- stacking is observed for **4**.

The absorptions features of **5** and **7** covering the visible region and significant ultraviolet and near-infrared wavelengths, have allowed us to include them among “black absorbers” of interest for OPV devices (see Figure 1).

As anticipated, the CT salts [Pt(Et_2_dazdt)_2_][Pt(mnt)_2_] (**8**), [Pt(Me_2_pipdt)_2_][Pt(mnt)_2_] (**9**), and [Pt(Me_2_pipdt)_2_][Pt(dtcr)_2_] (**10**) show the presence in the diffuse reflectance spectra of an additional band with respect to those of the components at 937 nm in **8**, 1270nm in **9** and 960 nm in **10**. In Figure 1, the reflectance spectrum of **9** is reported as being a representative example to show the absorptions in the near-IR region where the donor and acceptor molecules alone do not absorb.

Thus, according to the optical features of the adducts summarized above, it should be remarked that no growth of NIR peak is observed where adducts are based on donors displaying self-association.

For electrostatic reasons, self-association is prevented when the donor entity is represented by a dianionic bis-dithiolate, and the acceptor is represented by bicationic Pt(II) complexes, while it may be competing for neutral donors showing an asymmetric charge distribution such as in the diimine-dithiolate cases.

Another feature of paramount importance for both DACT and photovoltaic applications, is that related to the electrochemical properties of donors and acceptors (see Table 1). Concerning the CT, regardless of the packing motif, the more easily the donor is oxidized, and the acceptor reduced, the more important the DACT can be. However, for photovoltaic applications, in addition to suitable spectroscopic properties (absorption of light as intense and extended as possible) some electrochemical features are required. As an example, in the case of solar cell dyes, the molecular system (active medium) must present a first reduction peak a low potential (corresponding to an excited electronic state at higher energy than that of the conduction band of the semiconductor (SC)), as well as a first oxidation peak falling at higher potential then that of the redox electrolyte used in the cell. This allows an efficient electron transfer to the SC and the regeneration of the dye to its electronic ground state by reduction, respectively. The SC and the electrolyte usually employed are TiO_2_ and the couple I^−^/I_3_^−^, respectively; this requires reduction potentials more negative than −0.6 V and oxidation potentials more positive than +0.2 V. Analyzing the data reported in Table 1, it can be seen that whereas all the compounds fit the requirement for the oxidation potential, only **4**, which do not present any additional CT absorption, shows a first reduction peak at values lower than −0.6 V. However, a higher number of compounds (i.e., **5**, **8**–**10**, in addition to **4**) can be suitable for this kind of application if a SC with a conduction band at more positive energy value, such as SiO_2_ (−0.10 V), is used instead of TiO_2_.

### 2.2. Crystal Packing

Structural characterization for the **1**–**10** adducts allowed us to point out a variety of donor−acceptor stacking patterns in the solid state.

The systems considered in this work comprise square planar platinum complexes, either heteroleptic platinum-diimine-dithiolato neutral complexes, or dicationic homoleptic platinum-dithiolenes acting as donors stacked with entities acting as acceptors (Scheme 3). In complexes **1**–**7**, the acceptor is an organic molecule.

**Figure 2:**
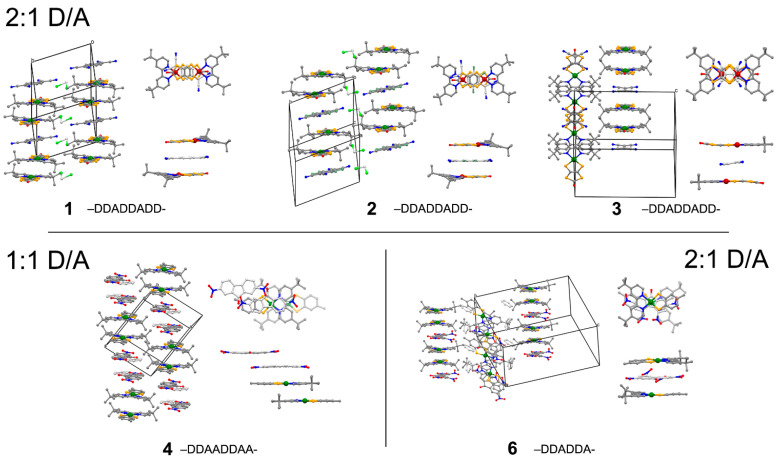
Top: Crystal packing of [Pt(dbbpy)(dmid)]_2_[TCNQ]·2CH_2_Cl_2_ (**1**) [Pt(dbbpy)(dmid)]_2_[TCNQF_4_]·2CH_2_Cl_2_ (**2**) and [Pt(dbbpy)(dmid)]_2_[TCNE]·C_6_H_6_ (**3**) [17]. All systems exhibit a columnar stack with a 2:1 D/A-DDADDADD-pattern. Two columnar stacks are depicted in the packing. Bottom, left: crystal packing of [Pt(dbbpy)(tdt)][DNF]·0.5C_6_H_6_ (**4**) having a columnar stack with a 2:2 D/A -DDAADDAADD- pattern. Two columnar stacks are depicted in the packing. Bottom, right: crystal packing of [Pt(dbbpy)(tdt)]_2_[TENF]·2C_6_H_6_ (**6**) having a columnar stack with a -DDADDADDA- pattern [11]. Three columnar stacks are depicted in the packing.

**Figure 3:**
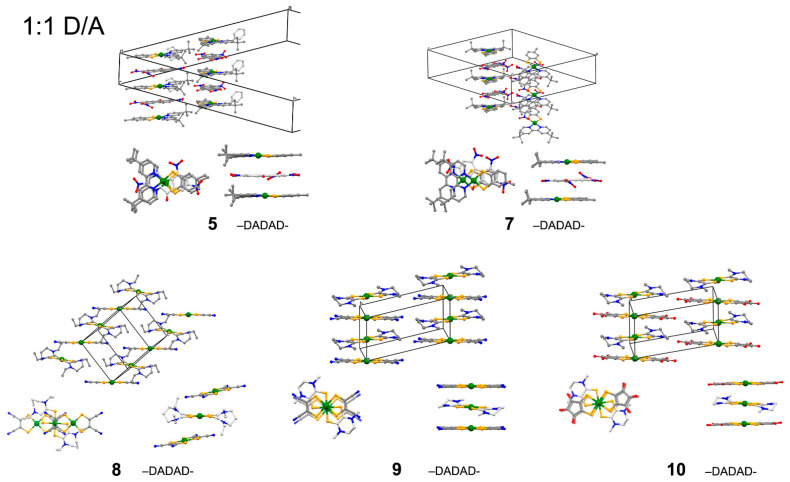
Top: crystal packing of [Pt(dbbpy)(tdt)][TRNF]·C_6_H_6_ (**5**) and [Pt(dbbpy)(tdt)][TENF]·0.5C_6_H_6_ (**7**) [11]. Bottom: crystal packing of [Pt(Et_2_dazdt)_2_][Pt(mnt)_2_] (**8**) [22], [Pt(Me_2_pipdt)_2_][Pt(mnt)_2_] (**9**) [23] and [Pt(Me_2_pipdt)_2_][Pt(dtcr)_2_] (**10**) [24]. All systems exhibit a columnar stack with a -DADAD- pattern. Two columnar stacks are depicted in the packing.

At variance, in **8**–**10**, the donor entity is represented by a dianionic bis-dithiolate Pt(II) complex, and the acceptor is represented by bicationic Pt(II) complexes. All systems form 1D columnar stacks, which differ by the type of stacking between the donor and acceptor. In particular, compounds **1**–**3** and **6** exhibit a 2:1 D/A ratio with a -DDADDADD- pattern, compounds **5**, **7** and **8**–**10** exhibit a 1:1 D/A ratio with a -DADAD- pattern, and compound **4** exhibits a 2:2 D/A ratio with a -DDAADDAADD- pattern. In all cases, solvent molecules of crystallization are present in the structure, and they are usually interposed between the columnar stacking. The molecular structures of the complexes and their crystal packings were previously reported [11,17]. Nevertheless, a brief description of the crystal packing is summarized here with the view to highlight the DA interactions. Even though the compounds exhibit different stacking patterns, their structural arrangement can be divided into four groups according to the stacking similarities. Group 1 includes compounds **1**, **2**, **3** and **6**, which exhibit a head-to-tail disposition of the donor D in both the -DAD- and in the -DD- sub-systems (Figure 2). Group 2 includes compounds **5** and **7**, which exhibit a head-to-head arrangement in the -DAD- system (see Figure 3). Moreover, in **7**, the presence of four nitro groups of the TENF molecule correspond to a larger steric hindrance with respect to the TRNF ligand. This implies that in **7**, the acceptor TENF is slightly offside with respect to the stacking of the D functions. Compound **4** (group 3) has peculiar features since it is characterized by a 2:2 -DDAA- stacking (see Figure 2).

In particular, the donors are in a head-to-tail arrangement in the -DD- sub-system, whereas one of the acceptors is overlaid on one of the D molecules, and the second acceptor is only partially stacked over the first one, with minimum contact between the central C=O group and a peripheral NO_2_ moiety. Finally, group 4 comprises complexes **8**–**10** whose stacking is shown in Figure 3. In more detail, **9** and **10** have a very similar pattern, since the planar PtS_4_ systems of both the donor and of the acceptor are nearly parallel; they are rotated with respect to each other by approximately 45° even though the metal atoms are aligned and stacked above each other. In **8**, the greater steric hindrance of the acceptor implies that the D and A moieties are not parallel, and the two interacting systems are offside with the metal atoms interacting with the periphery of the ligands.

### 2.3. Hirshfield Surface Analysis

**Figure 4:**
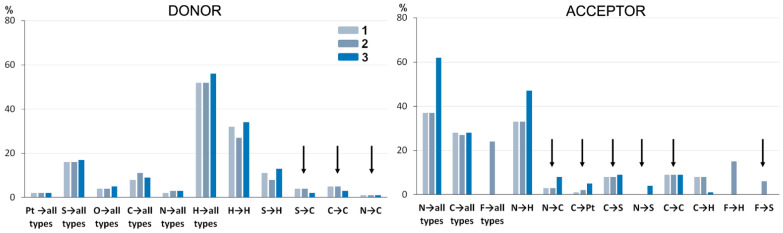
Percentage contributions of the various intermolecular contacts exchanged by the donor moiety and by the acceptor moiety with the surrounding environment for complexes **1**–**3** are reported as colored bars. The arrows indicate interactions occurring between atoms that are part of planar and electronically delocalized systems.

**Figure 5:**
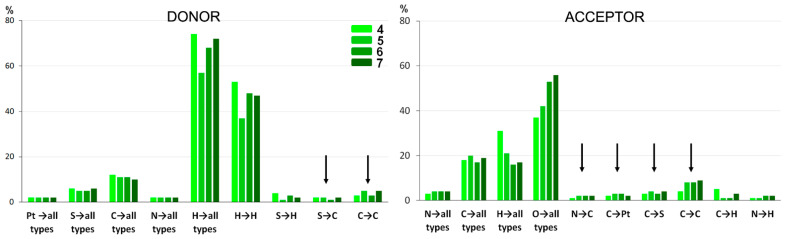
Percentage contributions of the various intermolecular contacts exchanged by the donor moiety and by the acceptor moiety with the surrounding environment for complexes **4**–**7** are reported as colored bars. The arrows indicate interactions occurring between atoms that are part of planar and electronically delocalized systems.

**Figure 6:**
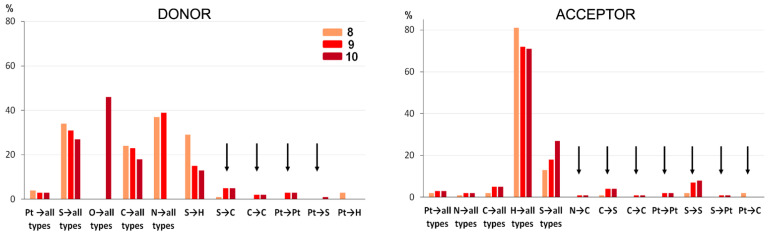
Percentage contribution of the various intermolecular contacts exchanged by the donor moiety and by the acceptor moiety with the surrounding environment for complexes **8**–**10** are reported as colored bars. The arrows indicate interactions occurring between atoms that are part of planar and electronically delocalized systems.

A comprehensive investigation of the different types of interactions exchanged by the D and A fragments in each one of the ten complexes under exam was performed by means of the Hirshfeld surface analysis [28,30]. In Figure 4, Figure 5 and Figure 6, the main types of interactions for the donor and the acceptor in each of the ten compounds under investigation are summarized. The comparison has been performed by grouping the compounds according to a similarity criterion involving both the donor and the acceptor. In particular, Figure 4 reports the interactions exchanged by the [Pt(dbbpy)(dmid)] donor and the TCNQ, TCNQF and TCNE acceptors, whereas Figure 5 illustrates the interactions exchanged by the [Pt(dbbpy)(tdt)] donor and the DNF, TRNF and TENF acceptors. In addition, with TENF, two different solvates were reported, which differed by the composition, stacking and solvent (C_6_H_6_) content in the crystal lattice. In Figure 6, the interactions exchanged by the [Pt(mnt)_2_]^2−^ and [Pt(dtcr)_2_]^2−^ donors and the [Pt(Et_2_dadzt)_2_]^2+^ and [Pt(Me_2_pipdt)_2_]^2+^ acceptors [31] are depicted.

The interactions of **1**–**3** are quite similar, and they reflect the similarities between the crystal packing of the three compounds. In particular, the donor exhibits an almost conserved interaction pattern, whereas the different molecular structures of the acceptors imply a more varied interaction scheme. The major types of interaction for the donor involve the H atoms, the sulphur atoms and, to a minor extent, the C, O and Pt atoms. In the acceptors, the interactions are dominated by the nitrogen atoms, which mainly interact with the H atoms of surrounding molecules. The C atoms are also involved in a good portion of the interactions, which involves Pt, S, and C atoms of the surrounding molecules. The different molecular structure of the acceptor TCNE with respect to TCNQ and TCNQF is reflected in the dominant contribution of the nitrogen atom in **3** with respect to the other types.

The compounds **4**–**7** differ in the number of nitro groups present on the acceptor molecules. As pointed out above, this heavily affects the structural arrangement since more varied crystal packing were observed. Nevertheless, according to the fragmental composition there is an almost preserved interactions pattern among the four compounds.

The main difference is observed for the interactions exchanged by the donor in **5**, in which there is a minor contribution deriving by the H atoms. The large contribution deriving by the H atoms in **1**–**7** is a consequence of the presence of *t*-butyl residues of dbbpy in the donor. This contribution is augmented in **4**–**7**, according to the presence of the tdt fragment. A minor but significant portion of the interactions in the donors of **4**–**7** are those involving the C, S and, to a lesser extent, the Pt atoms. The acceptors of **4**–**7** show a trend which is in line with the increasing number of nitro groups along the series, and the O atoms in **6** and **7** dominates the interactions.

The HS analysis on complexes **8**–**10** was already reported [22], and it is briefly summarized here for comparison purposes with those of compounds **1**–**7**. According to the presence of the PtS_4_ system for both the donor and the acceptor entity, in **8**–**10**, one of the dominant interactions is that involving the sulphur atoms.

In addition, a considerable fraction of the interactions is exchanged by the N atoms in **8** and **9** and by the O atoms in **10**, and this reflects the different molecular structure of the mnt and dtcr ligands. The C atoms of the donor are also involved in a good portion of the interactions. In the acceptor, the main type of interaction is that involving the H atoms followed by the S atoms. A distinctive feature that characterizes compound **9** and **10** with respect to the other ones is the presence of the Pt–Pt interaction. This is a consequence of the alignment of the D and A moieties in the two complexes. The greater steric hindrance of Et_2_dadzt in **8**, when compared to Me_2_pipdt in **9** and **10**, limits the overlap between the molecular planes of A and D, and in particular, it has the effect of hindering the approach of the platinum atoms. In fact, in **8**, the Pt atom of the acceptor is overlaid with the C-C fragment of the donor. In Figure 4, Figure 5 and Figure 6, the interactions that occurs between D and A are highlighted.

## 3. Materials and Methods

CrystalExplorer 3.1 program [32] was used to assess the different type of interactions in the solid state for the various compounds. Hirshfield surface (HS) analysis was performed for all of the systems. The HS defines the volume of space in a crystal where the sum of the electron density of spherical atom for the molecule (pro-molecule) exceeds that for the crystal (pro-crystal). Different properties of the HS can be computed and graphically visualized, in particular, *d_e_* and *d_i_*, which represent the distance from a point on the surface to the nearest nucleus outside or inside the surface, respectively. The *d_norm_* is the normalized contact distance, and it is defined by taking into account *d_e_* and *d_i_* and the van der Waals radii of the atoms:

(1) $$d_{norm}=\frac{d_{i}-r_{i}^{\nu dW}}{r_{i}^{\nu dW}}+\frac{d_{e}-r_{e}^{\nu dW}}{r_{e}^{\nu dW}}$$

*d_norm_* can be mapped on the HS, providing clear evidence of the interactions exchanged by adjacent molecular fragment. Moreover, the correlation between *d_e_* and *d_i_* provides the fingerprint plots, which are 2D diagrams that provide a thorough depiction of the overall interactions exchanged by the molecules within the crystal [33]. The fingerprint plots can provide the contribution of pairs of interacting atoms to the crystal packing. It is then possible to express the atom–atom interaction as a percentage of the overall interactions exchanged by the molecules within the crystal.

## 4. Conclusions

In the present work, we have analysed the stacking and interaction patters of ten D-A charge-transfer compounds consisting of donors based on platinum-dithiolene complexes and of acceptors based on tetranitrile, nitrofluorenone and platinum-dithiolene system, as a case study.

All the compounds of known crystal structures exhibit columnar stack between D and A and the following stacking patterns are observed: 1:1 -DA- for complexes **5**, **7**–**10**; 2:1 -DDA- for complexes **1**–**3**, **6**; 2:2 -DDAA- for complex **4**. The 1:1 -DA- stacking patterns alone (**5**, **7**, **8**, **9** and **10)** have been shown to be suitable to give rise to the NIR CT transition.

As a general observation, and according to the HS analysis, it is evident that the H atoms are responsible for a large portion of the interactions exchanged by the donor in **1**–**7** and by the acceptor in **8**–**10**. This is a consequence of the functionalization with t-butyl, ethyl or methyl groups on the Bipy, Et_2_dazdt and Me_2_pipdt ligands, respectively. Moreover, the acceptor of **1**–**7** is an organic moiety, and the main source of interactions is provided by N (**1**–**3**) and O (**4**–**7**) atoms.

It is reasonable to assume that the electronic communication between the donor and acceptor moieties leading to DACT transition (**5**, **7**, **8**, **9** and **10**) is more efficient between electronically delocalized molecular fragments in D and A. In this respect, by analysing the HS results, it is evident that the charge transfer may occur through a minor fraction of the entire group of interactions exchanged by the D or A systems. An even more minor fraction is that involving the platinum atoms. Notwithstanding the limited fraction of these interactions, they play a crucial role in the charge transfer properties and absorption features of this class of DA compounds. Moreover, the A + D → A^−^ + D^+^ CT process usually implies an electronic and structural reorganization of the A^−^ and D^+^ components with respect to the parent A/D system. Thus, the structural analysis discussed here for **1**–**10**, can describe the structural features of the initial A/D state but cannot describe the A^−^/D^+^ one, which is not experimentally accessible. Nevertheless, the decomposition of the A/D system into different interacting fragments provides additional hints on the molecular components that can favour the CT process.

## Figures and Tables

**Table 1:** Summary of packing motifs, donors and acceptor redox potentials and CT transition positions for the investigated compounds.

Compound	Packing Motif	D oxidation Potential (V) E_1/2_	A reduction Potential (V) E_1/2_	λ_max_ CT (nm)
**1**	-DDADD-	+0.54	+0.19	800–1500 broad-week abs
**2**	-DDADD-	+0.59	+0.59	“800–1500 broad-week abs
**3**	-DDADD-	+0.48	+0.17	800–1500 broad-week abs
**4**	-DDAADD-	+0.49 (E_p_)	−0.70	Not observed
**5**	-DADAD-	+0.50 (E_p_)	−0.42	900
**6**	-DDADD-	+0.48 (E_p_)	+0.18	Not available
**7**	-DADAD-	+0.48 (E_p_)	+0.18	950
**8**	-DADAD-	+0.21	−0.24	937
**9**	-DADAD-	+0.21	−0.13	1270
**10**	-DADAD-	+0.40	−0.15	960

## Data Availability

Not applicable.
